# *KCNQ1* rs2237892 C→T gene polymorphism and type 2 diabetes mellitus in the Asian population: a meta-analysis of 15,736 patients

**DOI:** 10.1111/jcmm.12185

**Published:** 2013-12-24

**Authors:** Yan-yan Li, Xiang-ming Wang, Xin-zheng Lu

**Affiliations:** a Department of Geriatrics, First Affiliated Hospital of Nanjing Medical UniversityNanjing, China; b Department of Cardiology, First Affiliated Hospital of Nanjing Medical UniversityNanjing, China

**Keywords:** *KCNQ1*, rs2237892, polymorphism, type 2 diabetes mellitus, Asian

## Abstract

The *KCNQ1* rs2237892 C→T gene polymorphism is reportedly associated with T2DM susceptibility, but various studies show conflicting results. To explore this association in the Asian population, a meta-analysis of 15,736 patients from 10 individual studies was performed. The pooled odds ratios (ORs) and their 95% confidence intervals (CIs) were evaluated using random-effect or fixed-effect models. A significant relationship between the *KCNQ1* rs2237892 C→T gene polymorphism and T2DM was observed in the Asian population under the allelic (OR, 1.350; 95% CI, 1.240–1.480; *P* < 0.00001), recessive (OR: 0.650; 95% CI: 0.570–0.730; *P* < 0.00001), dominant (OR: 1.450; 95% CI: 1.286–1.634; *P* < 0.00001), and additive (OR: 1.346; 95% CI: 1.275–1.422; *P* < 0.00001) genetic models. In the subgroup analysis by race, a significant association was found in Chinese, Korean and Malaysia population, but not in Indian population. *KCNQ1* rs2237892 C→T gene polymorphism was found to be significantly associated with increased T2DM risk in the Asian population, except Indian population. The C allele of the *KCNQ1* rs2237892 C→T gene polymorphism may confer susceptibility to T2DM.

## Introduction

Diabetes mellitus (DM) has become a major public health problem in China. About 9.7% of Chinese adults currently suffer from DM, which is not even diagnosed in 60.7% of these cases. Moreover, 15.5% of Chinese adults have pre-diabetes, which is an important risk factor for the progression of overt DM and cardiovascular diseases 1. Diabetes mellitus is clinically characterized by chronic hyperglycaemia accompanied by insulin defects both in secretion and action, which contribute to the metabolic disturbance of carbohydrate, lipid, and protein levels in the body, and the chronic impairment and dysfunction of several organs. In 1999, the World Health Organization (WHO) classified DM into four types: type 1 diabetes mellitus (T1DM), type 2 diabetes mellitus (T2DM), other special types and gestational DM. Among the four classes, T2DM accounts for more than 90% of the total cases. However, the aetiology or pathogenesis of T2DM has yet to be completely elucidated. Type 2 diabetes mellitus is generally considered to be a multifactorial disease influenced by the combined effects of heredity, behaviour and environment factors.

Type 2 diabetes mellitus is a polygenic metabolic disorder with genetic heterogeneity. The voltage-gated potassium channel or KQT-like subfamily member 1 (*KCNQ1*) gene has been identified as a novel gene susceptible to T2DM in the Genome-Wide Association Studies (GWAS) by Unoki *et al*. 2 in Japan. At the same time, Yasuda *et al*. 3 found that the association of *KCNQ1* with T2DM was replicated in populations with Korean, Chinese and European ancestry as well as in two independent Japanese populations.

KCNQ1 consists of 676 amino acids with six transmembrane regions and one ion-selective P loop. Four identical α subunits comprise the P loop, which is an ion-filter duct. It has a porous structure that is highly conservative, which confers its high selectivity to potassium.

*KCNQ1* gene, which is highly expressed in the heart, pancreas, inner ear stria vascularis, prostate, kidney, small intestine and peripheral blood leucocytes, is located in 11p15.5, spans 404 kb, and contains 17 exons. The *KCNQ1* rs2237892 locus point mutation in the 15th intron is the cytosine (C) that is being substituted with thymine (T).

Although many studies on the relationship between *KCNQ1* rs2237892 C→T gene polymorphism and T2DM have been performed, the results of the individual studies were inconsistent. Lee *et al*. 4 reported that the C allele of *KCNQ1* rs2237892 C→T gene polymorphism conferred a risk of T2DM in the Korean population. Similarly, Liu *et al*. 5 confirmed the effects of *KCNQ1* rs2237892 C variants in a Chinese population. By contrast, Chen *et al*. 6 did not find any difference in the prevalence of T2DM between the three genotypes of *KCNQ1* rs2237892 gene locus in another Chinese population.

As the genotype distribution is different between continents, this study involved the meta-analysis of 7607 T2DM patients and 8129 controls to determine the relationship of *KCNQ1* rs2237892 C→T gene polymorphism with T2DM in the Asian population (Data S1).

## Materials and methods

### Publication search and inclusion criteria

The electronic databases as PubMed, Embase, Web of Science, China National Knowledge Infrastructure, and China Biological Medicine Database were searched using the terms ‘*KCNQ1*’, ‘rs2237892’, ‘T2DM’, and ‘polymorphism’. The last research was updated on 10 September 2013, with the date of publication ranging from 2008 to 2012.

The selected studies had to meet the major criteria: (*i*) Evaluation of the *KCNQ1* rs2237892 C→T gene polymorphism and T2DM; (*ii*) the diagnosis of T2DM was according to the American Diabetes Association fasting plasma criteria (2005), which requires that the fasting plasma glucose levels of patients be ≥7.0 mmol/l or that the 2 hrs plasma glucose level be ≥11.1 mmol/l; (*iii*) the studies should be case–control or cohort studies published in the official journals; and (*iv*) the studies should be consistent with the Hardy–Weinberg equilibrium (HWE).

### Data extraction

The data were extracted with the use of a standard protocol. Three researchers performed the meta-analysis, two of whom found the parallel studies, while the third served as the arbitrator in case disagreements occur between the two researchers. The studies that were not in accord with the inclusion criteria, published repeatedly, or supplied insufficient data were excluded. If the same data resulted from different studies, the data were only adopted once. The extracted data comprised the following items: the first author's name, publication year, region, number of genotypes, genotyping, study design, matching criteria and the total number of cases and controls.

### Statistical analyses

Four genetic models, which include the allelic (distribution of C allelic frequency of *KCNQ1* rs2237892 C→T gene polymorphism), the recessive (TT *versus* CC+CT), the dominant (CC *versus* CT+TT) and the additive (C *versus* T) genetic models, were used. The association of *KCNQ1* rs2237892 C→T gene polymorphism and T2DM was compared by using ORs and their corresponding 95% CIs. The heterogeneity between the individual studies was calculated using Chi-square-based Q-tests and the significance level was set at *P* < 0.05 20. If the heterogeneity existed among the individual studies, the pooled OR was assessed using random-effect model (DerSimonian and Laird method) 21, otherwise, the fixed-effect model was adopted (the Mantel–Haenszel method) 22. The Z-test was used to determine the pooled OR and the significance level was set at *P* < 0.05.

The HWE was evaluated using Fisher's exact test, and the significance level was set at *P* < 0.05. The potential publication bias was estimated using funnel plot. The funnel plot asymmetry was assessed using Egger's linear regression test on the natural logarithm scale of the OR, and the significance level was also set at *P* < 0.05 23. The statistical analyses were performed with STATA 11.0 software (StataCorp, College Station, TX, USA).

## Results

### Studies and populations

Twenty-one related literatures were found. Among these were nine papers that were suitable to the inclusion criteria. Among the 12 excluded studies, one paper was a repeated publication, four were reviews and six were not involved with *KCNQ1* rs2237892 C→T gene polymorphism or T2DM. One study was performed in a European population and was rejected. No study was excluded for deviating from the HWE. The data were obtained from 7607 T2DM cases and 8129 controls (Table 1, Data S2) 4–12. The countries that were involved such as China, Korea, India and Malaysia are from Asia.

**Table 1:** Characteristics of the investigated studies of the association of *KCNQ1* rs2237892 C>T gene polymorphism and type 2 diabetes mellitus (T2DM) in the Asian population

			T2DM			Control				
Author	Year	Region	CC	CT	TT	CC	CT	TT	Matching criteria	Sample size (T2DM/control)
Lee 4	2008	Korea	389	377	99	182	239	75	Sex, ethnicity	865/496
Hu 7	2009	China	947	643	129	706	816	198	Ethnicity	1719/1720
Liu 5	2009	China	902	813	165	853	919	224	Ethnicity	1880/1996
Chen 6	2010	China	27	24	6	162	144	35	Ethnicity	57/341
Han 8	2010	China	525	396	69	415	437	107	BMI, ethnicity	990/959
Xu 9	2010	China	30	33	3	272	300	80	Ethnicity	66/652
Been 10	2011	India	1259	30	1	982	36	1	Ethnicity	1290/1019
Been 10	2011	US-Indian	133	6	0	523	32	2	Ethnicity	139/557
Saif-Ali 11	2011	Malaysia	135	79	20	81	75	21	Sex, ethnicity	234177
Dai 12	2012	China	233	112	22	110	82	20	Age,BMI, ethnicity	367/212

T2DM: type 2 diabetes mellitus; BMI: body mass index.

Polymerase chain reaction (PCR) genotyping method and case–control study design have been adopted in all of the above studies.

US-Indian: the Indian migrants living in the United States.

### Pooled analyses

**Fig 1:**
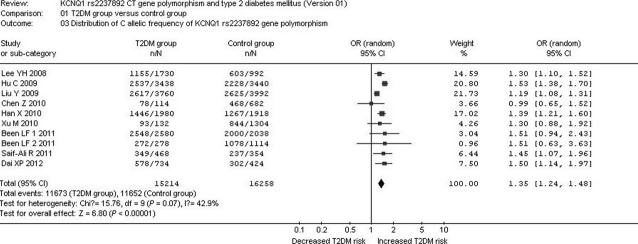
Forest plot of T2DM associated with *KCNQ1* rs2237892 CT gene polymorphism under an allelic genetic model (distribution of C allelic frequency of *KCNQ1* rs2237892 gene).

**Fig 2:**
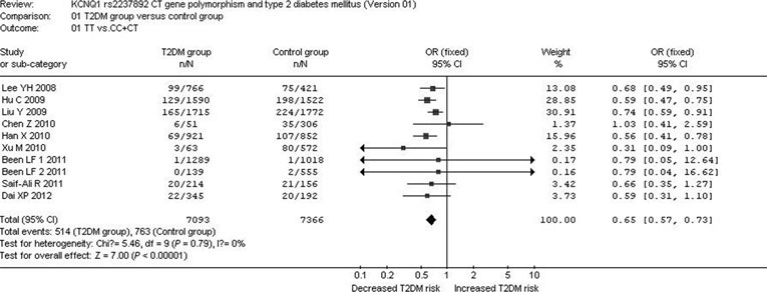
Forest plot of T2DM associated with *KCNQ1* rs2237892 CT gene polymorphism under a recessive genetic model (TT *versus* CC+CT).

A significant relationship between *KCNQ1* rs2237892 C→T gene polymorphism and T2DM was observed in the Asian population under the allelic (OR: 1.350; 95% CI: 1.240–1.480; *P* < 0.00001), recessive (OR: 0.650; 95% CI: 0.570–0.730; *P* < 0.00001), dominant (OR: 1.450; 95% CI: 1.286–1.634; *P* < 0.00001), and additive genetic models (OR: 1.346; 95% CI: 1.275–1.422; *P* < 0.00001; Table 2, Figs 1 and 2).

**Table 2:** Summary of meta-analysis of association of *KCNQ1* rs2237892 C>T gene polymorphism and type 2 diabetes mellitus (T2DM) in the Asian population

Genetic model	Pooled OR (95% CI)	*P*-value	Study number	T2DM size	Control size	*P*_heterogeneity(*I2%*)_
Allelic genetic model	1.350 (1.240–1.480)	<0.00001*	10	7607	8129	0.07 (42.9%)
Recessive genetic model	0.650 (0.570–0.730)	<0.00001*	10	7607	8129	0.79 (0%)
Dominant genetic model	1.450 (1.286–1.634)	<0.00001*	10	7607	8129	0.044* (48.1%)
Subgroup 1: T0 < 600	1.445 (1.231–1.696)	<0.00001*	5	1662	1783	0.654 (0%)
Subgroup 2: T0 > 600	1.449 (1.206–1.739)	<0.00001*	5	5945	6346	0.005* (73.1%)
Additive genetic model	1.346 (1.275–1.422)	<0.00001*	10	7607	8129	0.072 (42.9%)

* *P* < 0.05.

T2DM: type 2 diabetes mellitus; CI: confidence interval; OR: odds ratio; T2DM size: the total number of T2DM cases; control size: the total number of control group; Allelic genetic model: G allele distribution frequency; recessive genetic model: TT *versus* CC+CT; Dominant genetic model: CC *versus* CT+TT; Additive genetic model: total C allele *versus* total T allele.

Significant heterogeneity existed under the dominant genetic model (*P*_heterogeneity_ = 0.044, *I*^2^ = 48.1%). The subsequent meta-regression was carried out to explore the source of the heterogeneity. Under the dominant genetic model, the heterogeneity could be explained by CC genotype number of the T2DM group sample size (CC1, *P* = 0.005), total T2DM group sample size (T1, *P* = 0.001), CT genotype number of the T2DM group (CT1, *P* = 0.004), TT genotype number of the T2DM group (TT1, *P* = 0.003), CC genotype number of the control group sample size (CC0, *P* = 0.001) and CT genotype number of the control group sample size (CT0, *P* = 0.001). Based on T0, the whole population was divided into two subgroups. The studies with T0 < 600 belonged to subgroup 1 and the remaining studies with T0 > 600 belonged to subgroup 2. In the following subgroup analysis stratified by T0, significant T2DM risk increases were detected in the two subgroups (subgroup 1: OR: 1.445; 95% CI: 1.231–1.696; *P* < 0.00001; subgroup 2: OR: 1.449; 95% CI: 1.206–1.739; *P* < 0.00001). Nevertheless, the heterogeneity was significantly reduced in subgroup 1 (*P*_heterogeneity_ = 0.654, *I*^2^ = 0%) and was even higher in subgroup 2 than in the entire population (*P*_heterogeneity_ = 0.005, *I*^2^ = 73.1%) (Tables 2 and 3).

**Table 3:** The meta-regression results among 10 studies under the dominant genetic model for the association of *KCNQ1* rs2237892 C>T gene polymorphism and type 2 diabetes mellitus (T2DM) in the Asian population

	Coefficient	Standard Error	T-value	*P*-value	95% Confidence Interval
CC1	0.0002736	0.0000363	7.54	0.005*	0.0001581 ∼ 0.0003891
T0	−0.0219024	0.001866	−11.74	0.001*	−0.027841 ∼ −0.0159639
CT1	−0.0025551	0.0003243	−7.88	0.004*	−0.0035871 ∼ −0.0015231
TT1	0.0111866	0.0013193	8.48	0.003*	0.006988 ∼ 0.0153853
CC0	0.0211946	0.0018378	11.53	0.001*	0.0153457 ∼ 0.0270434
CT0	0.0278568	0.0023539	11.83	0.001*	0.0203657 ∼ 0.0353479
_cons	0.2189861	0.012387	17.68	0.000	0.179565 ∼ 0.2584071

* *P* < 0.05.

Coefficient: regression coefficient.

The regression coefficients are the estimated increase in the lnOR per unit increase in the covariates.

Cons, constant item; TT1, TT genotype number of T2DM group sample size; CC1, CC genotype number of T2DM group sample size; CT0, CT genotype number of control group sample size; CC0, CC genotype number of control group sample size; T0, total control group sample size.

In the subgroup analysis stratified by race, a significant association between *KCNQ1* rs2237892 C→T gene polymorphism and T2DM was observed in the Chinese subgroup under the allelic (OR: 1.350; 95% CI: 1.270–1.430; *P* < 0.00001), recessive (OR: 0.640; 95% CI: 0.560–0.730; *P* < 0.00001), dominant (OR: 1.424; 95% CI: 1.198–1.692; *P* < 0.00001), and additive genetic models (OR: 1.344; 95% CI: 1.187–1.522; *P* < 0.00001). A significant association was also found between *KCNQ1* rs2237892 C→T gene polymorphism and T2DM in the Korean subgroup under the allelic (OR: 1.300; 95% CI: 1.100–1.520; *P* = 0.002), recessive (OR: 0.680; 95% CI: 0.490–0.950; *P* = 0.02), dominant (OR: 1.410; 95% CI: 1.124–1.768; *P* = 0.003), and additive genetic models (OR: 1.296; 95% CI: 1.102–1.524; *P* = 0.002). In the Malaysian subgroup, a significant association was also observed under the allelic (OR: 1.450; 95% CI: 1.070–1.960; *P* = 0.02), dominant (OR: 1.616; 95% CI: 1.091–2.395; *P* = 0.017) and additive genetic models (OR: 1.448; 95% CI: 1.068–1.962; *P* = 0.017). However, no significant association was found between *KCNQ1* rs2237892 C→T gene polymorphism and T2DM under the recessive genetic model (OR: 0.660; 95% CI: 0.350–1.270; *P* = 0.22).

**Fig 3:**
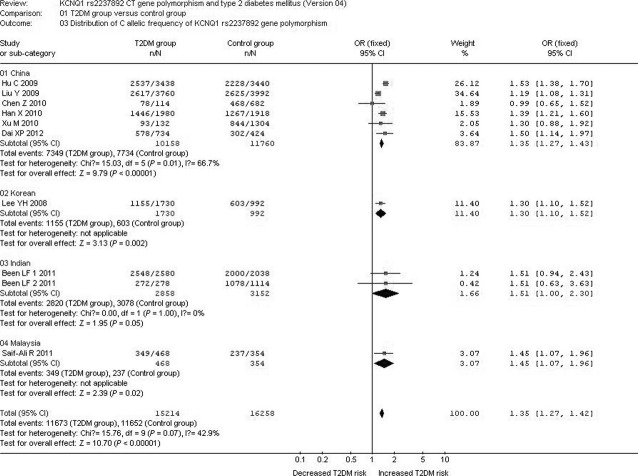
Forest plot of T2DM associated with *KCNQ1* rs2237892 CT gene polymorphism stratified by races under an allelic genetic model (distribution of C allelic frequency of *KCNQ1* rs2237892 gene).

**Fig 4:**
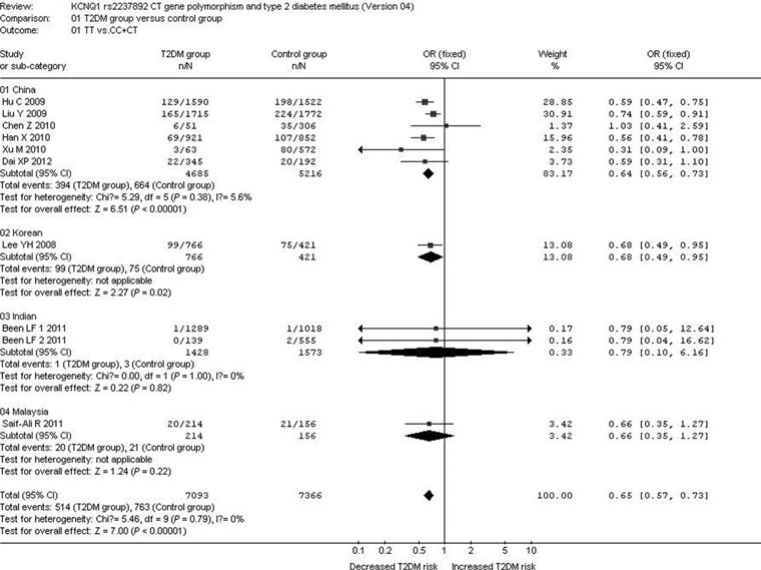
Forest plot of T2DM associated with *KCNQ1* rs2237892 CT gene polymorphism stratified by races under a recessive genetic model (TT *versus* CC+CT).

With regard to the Indian subgroup, no significant association was found between *KCNQ1* rs2237892 C→T gene polymorphism and T2DM under the allelic (OR: 1.510; 95% CI: 1.000–2.300; *P* = 0.05), recessive (OR: 0.790; 95% CI: 0.100–6.160; *P* = 0.82), dominant (OR: 1.509; 95% CI: 0.986–2.309; *P* = 0.058) and additive genetic models (OR: 1.513; 95% CI: 0.998–2.295; *P* = 0.051) (Table 4, Figs 3 and 4).

**Table 4:** Summary of meta-analysis of association of *KCNQ1* rs2237892 C>T gene polymorphism and type 2 diabetes mellitus (T2DM) stratified by race in the Asian population

Genetic model	Pooled OR (95% CI)	*P*-value	Study number	T2DM size	Control size	*P*_heterogeneity(*I2%*)_
Allelic genetic model	1.350 (1.270–1.420)	<0.00001*	10	7607	8129	0.07 (42.9%)
Chinese subgroup	1.350 (1.270–1.430)	<0.00001*	6	5079	5880	0.01 (66.7%)
Korean subgroup	1.300 (1.100–1.520)	0.002*	1	865	496	NA
Indian subgroup	1.510 (1.000–2.300)	0.05	2	1429	1576	1.0 (0%)
Malaysian subgroup	1.450 (1.070–1.960)	0.02*	1	234	177	NA
Recessive genetic model	0.650 (0.570–0.730)	<0.00001*	10	7607	8129	0.79 (0%)
Chinese subgroup	0.640 (0.560–0.730)	<0.00001*	6	5079	5880	0.38 (5.6%)
Korean subgroup	0.680 (0.490–0.950)	0.02*	1	865	496	NA
Indian subgroup	0.790 (0.100–6.160)	0.82	2	1429	1576	1.0 (0%)
Malaysian subgroup	0.660 (0.350–1.270)	0.22	1	234	177	NA
Dominant genetic model	1.450 (1.286–1.634)	<0.00001*	10	7607	8129	<0.044* (48.1%)
Chinese subgroup	1.424 (1.198–1.692)	<0.00001*	6	5079	5880	0.005 (70.5%)
Korean subgroup	1.410 (1.124–1.768)	0.003*	1	865	496	NA
Indian subgroup	1.509 (0.986–2.309)	0.058	2	1429	1576	0.907 (0%)
Malaysian subgroup	1.616 (1.091–2.395)	0.017*	1	234	177	NA
Additive genetic model	1.346 (1.275–1.422)	<0.00001*	10	7607	8129	<0.072 (42.9%)
Chinese subgroup	1.344 (1.187–1.522)	<0.00001*	6	5079	5880	0.01 (66.7%)
Korean subgroup	1.296 (1.102–1.524)	0.002*	1	865	496	NA
Indian subgroup	1.513 (0.998–2.295)	0.051	2	1429	1576	0.999 (0%)
Malaysian subgroup	1.448 (1.068–1.962)	0.017*	1	234	177	NA

* *P* < 0.05.

A logistic regression on multivariable adjusted risks such as age, sex, body mass index (BMI), ethnicity and sample size have been carried out. These risk factors were found to have no effect on the association of *KCNQ1* rs2237892 C→T gene polymorphism and T2DM (OR = 1).

### Diagnostics bias

**Fig 5:**
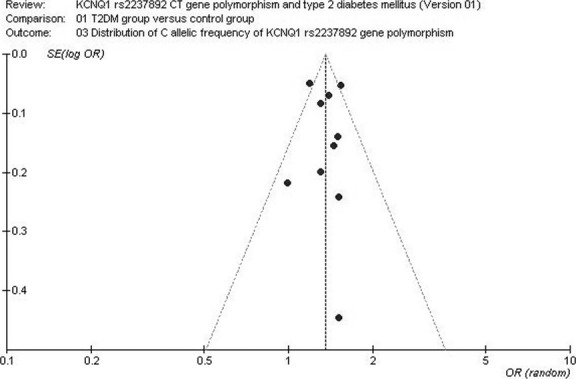
Funnel plot for studies of the association of T2DM associated with *KCNQ1* rs2237892 CT gene polymorphism under an allelic genetic model (distribution of C allelic frequency of *KCNQ1* rs2237892 gene). The horizontal and vertical axis correspond to the OR and confidence limits. OR: odds ratio; SE: standard error.

The publication bias of the individual studies was assessed using the funnel plot and Egger's test. No visual publication bias was found in the funnel plot (Fig. 5), and no significant difference was observed in Egger's test. Thus, no publication bias existed in the current meta-analysis (dominant genetic model, *T* = 1.27, *P* = 0.241).

## Discussion

In the current meta-analysis, a significant relationship existed between *KCNQ1* rs2237892 C→T gene polymorphism and T2DM under allelic (OR: 1.350), recessive (OR: 0.650), dominant (OR: 1.450) and additive genetic (OR: 1.346) models in the Asian population. In conclusion, the present results showed that the *KCNQ1* rs2237892 C allele may increase T2DM risk and confer T2DM susceptibility to Asians.

Taking into account that the heterogeneity existed under the dominant genetic model (P_heterogeneity_ < 0.05), the meta-regression has been carried out to find the heterogeneity source. In the subsequent heterogeneity source analysis under the dominant genetic model, T0 was shown to be the main possible heterogeneity source (*P* = 0.001), while in the additive genetic model, the confounding factor CC0 could explain the heterogeneity source (*P* = 0.008).

Under the dominant genetic model, the subgroup analysis stratified by T0 demonstrated that although increased T2DM risks were observed in both subgroups, the heterogeneity was weakened in the subgroup 1 (*I*^2^ = 0%) and strengthened in the subgroup 2 (*I*^2^ = 73.1%). Hence, T0 mainly caused the heterogeneity under the dominant genetic model. Moreover, the total control group sample size should be better matched between the individual studies to further reduce the heterogeneity.

In the subgroup analysis stratified by race, a significant relationship existed between the *KCNQ1* rs2237892 C→T gene polymorphism and T2DM in Chinese, Korean, and Malaysian subgroups but not in the Indian population. The varying results between the different races were probably associated with different ethnicities. The consequences of these racial differences might have on the results and conclusions. Additionally, the number of manuscripts included in the Korean, Indian and Malaysian subgroups was relatively small, which might have caused the difference among the races. More studies should be carried out in the future to verify the association between *KCNQ1* rs2237892 C→T gene polymorphism and T2DM in Asian populations.

At present, the exact mechanism of the association of *KCNQ1* rs2237892 C→T gene polymorphism and T2DM has not yet been elucidated. *KCNQ1* gene encodes for the pore-forming subunit of the voltage-dependent potassium ion channel, which is required for the repolarization of the cardiac action potential and for water and salt transportation in epithelial tissues. Previous studies have shown that *KCNQ1* variants led to cardiac long-QT syndrome and sudden infant death syndrome 13,14. For T2DM, *KCNQ1* is produced in the pancreatic islets, and the specific *KCNQ1* blocker 293B stimulates insulin secretion 15. In 2010, Zhou *et al*. 16 found that fasting plasma glucose concentration was associated with the C risk allele of rs2237892, suggesting that the baseline insulin secretion was impaired for the CC homozygote of rs2237892. The long-term survival rate of the pancreatic β cells has been suggested as depending on the condition of the potassium ion channel. The CC homozygote of rs2237892 might also influence the β cell function and insulin secretion, thus conferring increased susceptibility to T2DM. In 2009, Liu *et al*. 5 found that the *KCNQ1* gene might have an effect on T2DM through factors other than weight gain. In the current meta-analysis, the *KCNQ1* rs2237892 C allele increased the T2DM risk, which may be associated with insulin hyposecretion. However, this hypothesis needs to be verified by more studies.

In 2010, Zhou *et al*. 16 have performed a meta-analysis on the relationship between *KCNQ1* rs2237892 C→T gene polymorphism and T2DM, and they concluded that the *KCNQ1* rs2237892 C allele increased the T2DM risk. Although the conclusion was similar to that obtained from the current meta-analysis, the current meta-analysis was more superior as compared with their work. Zhou's paper was published in 2010, while the current meta-analysis included literature published from 2010 up to the present. In addition, only the dominant and recessive genetic models were adopted in Zhou's work, while in the current meta-analysis, four genetic models were used namely, the allelic, dominant, recessive and additive genetic models. Hence, the conclusion of this study must be more objective and credible than theirs. Meanwhile, Unoki *et al*. 2, Yasuda *et al*. 3, and Tan *et al*. 17 also examined the effects of single nucleotide polymorphisms in *KCNQ1* rs2237892 using genome-wide association studies and obtained positive results. However, concrete data were not available and cannot be adopted. Moreover, with regard to the study of Tan *et al*. 17 involving meta-analysis, only the allelic genetic model is used and the credibility of their results is doubtful. In addition, Sun *et al*. 18 and Liu *et al*. 19 also performed two meta-analyses on the relationship between *KCNQ1* rs2237892 C→T gene polymorphism and T2DM afterwards. Although they obtained positive results, the number of genotypes in the aforementioned manuscripts could not be retrieved yet and neither of their conclusions was credible. In the current meta-analysis, the data extraction process was stricter than theirs, and the results and the corresponding conclusion must be more objective than the previous meta-analyses. Furthermore, considering that the difference races might influence the research results, the subgroup analysis stratified by races have been performed in the current meta-analysis and it was shown that there was a significant association of T2DM with *KCNQ1* rs2237892 C→T gene polymorphism in Chinese, Korean and Malaysia population and not in the Indian population. However, in those previous meta-analyses, such subgroup analysis has not been carried out. Hence, the conclusion deduced from the present meta-analysis should be more comprehensive than those from the previous meta-analyses.

However, the present meta-analysis has some limitations. Large-scale studies on the association of T2DM with KCNQ1 rs2237892 C→T gene polymorphism were still not adequate. Although logistic regression has shown that age, sex, BMI, ethnicity, or sample size has no effect on the association of *KCNQ1* rs2237892 C→T gene polymorphism and T2DM in the current research, the KCNQ1 expression level was influenced by other genetic factors. As T2DM is a multigenic heredity disease, *KCNQ1* rs2237892 C→T gene polymorphism might be associated with the gene linkage disequilibrium of *KCNQ1* rs2237895 A→C, rs2237897 C→T and rs2074196 G→T gene polymorphisms, which might increase the T2DM risk 7.

*KCNQ1* rs2237892 C→T gene polymorphism was markedly associated with increased T2DM risk in the Asian population, except Indian population. People with the C allele might be susceptible to T2DM risks. The current conclusion might help us to formulate individual T2DM therapy strategies. Given the limitations, more large-scale studies are needed to clarify the significance of the conclusion.

## Supporting information

Data S1 PRISMA 2009 checklist.

Data S2 PRISMA 2009 flow diagram.
