# Supplementary material for: KCNQ1 rs2237892 C→T gene polymorphism and type 2 diabetes mellitus in the Asian population: a meta-analysis of 15,736 patients
Source: J Cell Mol Med. 2013 Dec 24;18(2):274–82. doi: 10.1111/jcmm.12185 (PMC3930414; doi:10.1111/jcmm.12185)
Supplement: Data S2 — PRISMA 2009 flow diagram. [file jcmm0018-0274-sd2.doc]

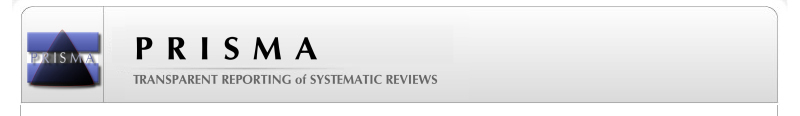
**PRISMA 2009 Flow Diagram**

**Screening**

**Included**

**Eligibility**

**Identification**

Records identified through database searching
(n =21 )

Additional records identified through other sources
(n =0 )

Records after duplicates removed
(n =20)

Records screened
(n =16 )

Records excluded for review characteristic
(n =4 )

Full-text articles assessed for eligibility
(n =16)

Full-text articles excluded for deviation from HWE (n =0 )

Studies assessed for eligibility
(n =10)
(n =9)

Records excluded for no association with KCNQ1 rs2237892 CT or T2DM

(n =6)

Studies included in qualitative synthesis
(n =9)

Records excluded for performed in Europe population (n = 1)

Records excluded for duplicated publication (n = 1)
